# Skull traction reattachment combined with ACDF in the treatment of C2-C3 transverse dislocation with unilateral cervical facet locked: A case report

**DOI:** 10.1016/j.ijscr.2025.111085

**Published:** 2025-02-24

**Authors:** Fan Yang, Jiongbiao Zhong, Zheng Li, Peishan Wang, Jiarui Peng, Qing Quan

**Affiliations:** Orthopedics Department, Yueyang Hospital Affiliated to Hunan Normal University, Yueyang 414000, Hunan Province, China

**Keywords:** Skull traction, ACDF, Intervertebral disc tear, C2-C3 transverse dislocation, Unilateral cervical facet locked

## Abstract

**Introduction and importance:**

When the cervical spine is subjected to flexion-rotation violence, it is easy to cause posterior cervical facet locked, with some patients a ‘tip-to-tip’ locked. Treatments are needed to perform, including reattaching the facet joint locked, dealing with the injured intervertebral disc, decompressing the spinal canal, and reconstructing the stability of the cervical spine. Each procedure is a challenge for the surgeon, so that there is no standardized surgical plan for these patients (Li et al., 2019 [1]). This case report described a typical patient who achieved a successful operation of ACDF after skull traction reattachment of the unilateral locking facet and C2-C3 transverse dislocation, which provided a reference for the surgical options for such patients.

**Case presentation:**

The patient was a 67-year-old male patient. His chief complaint was that six hours before arriving at the hospital, he suffered a car accident which caused his persistent neck pain with no effective measures of pain relief and his neck could not move. There were no such accompanying symptoms as nausea, vomiting, dizziness, and headache. Physical examination showed that the patient's limbs had no obvious limitation of movement and no symptoms of nervous injury. However, the right side of the patient's neck was significantly swollen, and the trachea was shifted to the left, The patient had no history of chronic diseases such as hypertension, diabetes, and coronary heart disease.

**Clinical discussion:**

For patients with single-segment cervical disc tear without neurological symptoms combined with vertebral body laterally shifted and unilateral ‘tip-to-tip’ type cervical facet locked, skull traction can safely and effectively reattach the locking cervical facet, especially for unilateral ‘tip-to-tip’ type (Rao et al., 2021; Hun et al., 2018; Davis et al., 1991 [7,10,11]). The following operation of ACDF alone can easily obtain satisfactory decompression of the spinal canal and stability reconstruction to the cervical spine.

**Conclusion:**

After successful reattachment of the locked cervical facet joint by skull traction, elective operation of ACDF was treated on the patient with single-segment cervical disc tear combined with C2-C3 transverse dislocation and unilateral ‘tip-to-tip’ facet locked. We found that this scheme can not only directly deal with the torn intervertebral disc, but also can effectively complete the decompression of the spinal canal and stability reconstruction of the cervical spine. The patient had a small operative incision and fewer postoperative complications. The early clinical efficacy of the patient was satisfactory.

## Highlights

• After successful reattachment of the locked cervical facet joint by skull traction, elective operation of ACDF was treated on the patient with single-segment cervical disc tear combined with C2-C3 transverse dislocation and unilateral 'tip-to-tip' facet locked.
• We found that this scheme can not only directly deal with the torn intervertebral disc, but also can effectively complete the decompression of spinal canal and stability reconstruction of cervical spine.
• The patient had a small operative incision and fewer postoperative complications. The early clinical efficacy of the patient was satisfactory.

## 1 Introduction

When the cervical spine is subjected to external flexion-rotation violence, it is easy to cause unilateral cervical facet locking, dislocation, and even cervical disc injury， because the posterior facet joint surface of the cervical vertebra is relatively flat. Some of these patients are ‘tip-to-tip’ type cervical facet locked. The main treatment strategy for these patients is to reattach the locked facet, and reconstruct the normal sequence of the cervical spine, and maintain the stability of the cervical spine [1]. However, its surgical treatment is still controversial. This report describes a case of a patient who does an elective operation of ACDF after skull traction. This scheme is used to treat patients with single-segment cervical disc tear without neurological symptoms combined with C2-C3 transverse dislocation and unilateral ‘tip-to-tip’ facet locked, which provides a reference for future surgical options for these patients. This case report was based on the SCARE criteria.

## 2 Case presentation

### 2.1 Case history

The patient was a 67-year-old male patient. His chief complaint was that six hours before arriving at the hospital, he suffered a car accident which caused his persistent neck pain with no effective measures of pain relief and his neck could not move. There were no such accompanying symptoms as nausea, vomiting, dizziness, and headache.

### 2.2 Physical examination

Physical examination showed that the patient's limbs had no obvious limitation of movement and no symptoms of nervous injury. However, the right side of the patient's neck was significantly swollen, and the trachea was shifted to the left, The patient had no history of chronic diseases such as hypertension, diabetes, and coronary heart disease.

### 2.3 Image analysis

**Fig. 1:**
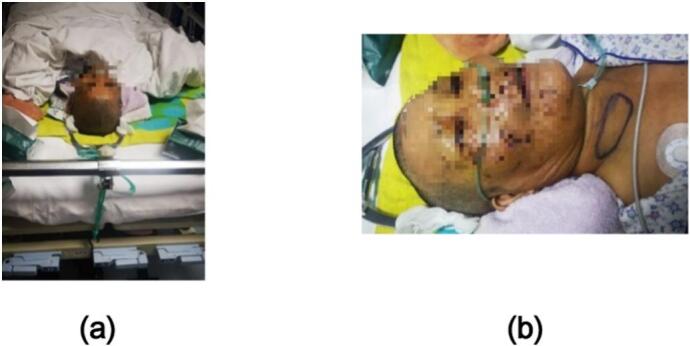
After admission, the patient was given skull traction at the bedside **(1a)**. At the same time, we found a hematoma in the patient's right neck **(1b)**. After continuous neutral traction, the facet joint was slightly flexed, and then laterally flexed to the opposite side to obtain a satisfactory reattachment of the facet joint.

The Neck Disability Index (NDI), JOA score, operation time, intraoperative blood loss, and perioperative complications were recorded before and after the operation. The improvement of the height of the injured intervertebral space, the situation of the reattachment of the facet joint, and the improvement of the Cobb angle of the C2–7 cervical lordosis (the acute angle formed by the vertical line of the lower edge of the C2 vertebral body and the vertical line of the lower edge of the C7 vertebral body, see 1b) before and after surgery was compared by imaging. The situation of interbody fusion was evaluated by imaging according to the modified Brantigan criteria [2]. (Fig. 1.)

### 2.4 Operation

**Fig. 2:**
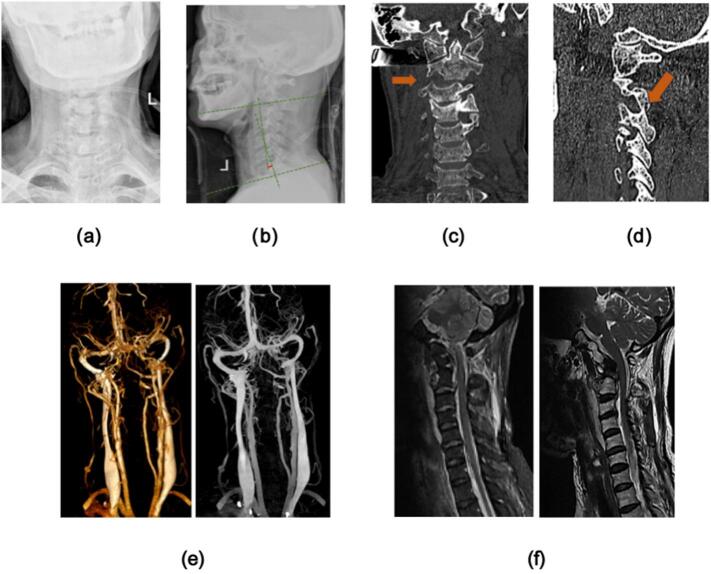
Preoperative cervical X-ray **(2a. 2b)**, CT **(2c, 2d)**, neck arterial-venous CTA **(2e)**, and MRI **(2f)** were performed showing C2-C3 transverse dislocation and facet joint locked, the arterial-venous of the neck was no obvious injury **(2e)**, and the Cobb angle of C2–7 cervical lordosis was measured to be 11°. MRI sagittal T2W1 showed C2–3 disc injury **(2f)**.

**Fig. 3:**
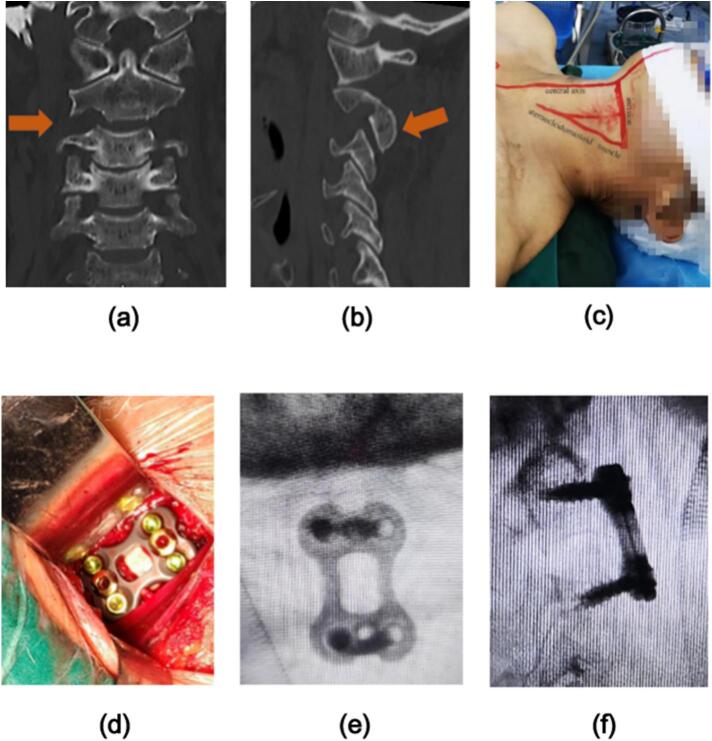
Before the operation, it was reconfirmed that the locking cervical facet was reattached **(3a, 3b)**. Because there was a hematoma on the right side of the patient's neck, the left anterior cervical transverse incision was performed to treat with the operation of ACDF on the injured segment **(3c, 3d)**. After the operation, it was confirmed that the internal fixation position was satisfactory and the physiological curvature of the cervical spine and the height of the intervertebral space were restored **(3e, 3f)**.

Skull traction was performed immediately after admission. The traction weight of the patient was 4–10 Kg, and the average traction weight was (6.0 ± 2.0) Kg. At the same time, the patient's neck arterial-venous CTA examination was immediately performed, the result showed that did not find obvious contrast agent leakage. Subsequently, we closely observed the patient's condition and gave the patient symptomatic treatment. After continuous skull traction, we reexamination the patient's neck CT again, it was confirmed that the locked facet joints of the patient had been reattached. With the patient's condition tended to be stable, an elective operation of ACDF was performed on the injured segment of the cervical spine, and intravenous inhalation combined with anesthesia was used during the operation. The patient was placed in a supine position, and a right anterior cervical transverse incision was made about 5 cm in length. The skin, subcutaneous tissue, and platysma were cut. The gap between the cervical vascular sheath and the visceral sheath was isolated, and the trachea esophagus and esophagus were pulled to expose the anterior fascia of the vertebra. The C-arm X-ray fluoroscopy was used to determine the intervertebral disc of the injured segment, and the target intervertebral disc was confirmed under direct vision. The outer annulus fibrosus was incised with a sharp knife to observe the deep intervertebral disc injury. A spatula and nucleus pulposus forceps were used to completely remove the injured intervertebral disc from the endplate. The bilateral uncinate joints were examined and bitten with a thin-mouth plier. Under the microscope, we carefully handled the posterior edge of the vertebral body to fully expose the posterior longitudinal ligament, then picked up the posterior longitudinal ligament with a thin hook and cut it with scissors to expose the cerebral dura mater, and fully decompress the head and tail. The cerebral dura mater and the nerve roots on both sides were explored and confirmed again without compression. The bone graft beds on both sides of the endplate were repaired and rinsed with normal saline, and the gelatin sponge was used to stop bleeding. The size of the intervertebral fusion cage was determined, and the Cage loaded with autologous bone particles was inserted into the intervertebral space. Through C-arm fluoroscopy, the Cage position was good. The appropriate length of the anterior cervical titanium plate was selected, and the titanium plate was locked by locking screws. The internal fixation position was determined to be satisfactory by fluoroscopy again. Finally, the incision was washed with physiological saline, and we put a drainage tube in the incision before closing the incision. (Fig. 2.Fig. 3).

### 2.5 Postoperative management

After the operation, antibiotics, hormones, and dehydration drugs were routinely used for 4–5 days, and atomization inhalation was performed for 3 days. The drainage tube was removed 48–72 h after operation. X-ray and CT were reexamined after the operation to understand the reduction of vertebral body and internal fixation. The patient was suggested to wear neck circumference external fixation for 3 months, and the function training of the cervical spine was performed according to the patient's recovery.

### 2.6 Follow-up/imaging

**Fig. 4:**
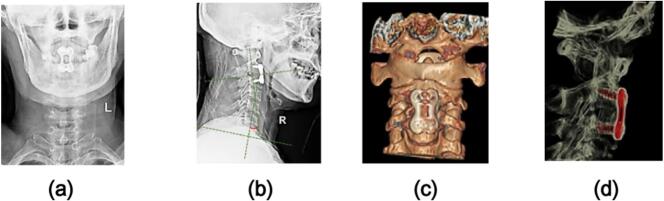
One year after operation, the patient was reexamined by cervical X-ray and CT(4a,4b,4c,4d). The Cobb angle of the injured C2-7 was improved by 16°, and the intervertebral height of the injured segment was restored by 1.8 mm compared with that at the time of injury.

The Neck Disability Index (NDI) score was 41 before the operation and 24 one year after the operation. The preoperative JOA score was 14, and the JOA score was 15 at 1 year after operation. The Cobb angle of C2–7 cervical lordosis was about 11° before the operation. The Cobb angle of C2–7 cervical lordosis was about 27° at 1 year after operation. The intervertebral height of the injured segment was about 2.6 mm before operation. The intervertebral height of the injured segment was about 4.4 mm at 1 year after operation. Compared with the preoperative image, the Cobb angle of C2–7 cervical lordosis, the intervertebral height of the injured segment, and the NDI score were significantly improved. (Fig. 4).

## 3 Clinical discussion

When the cervical spine is subjected to momentary flexion with rotation or lateral flexion violence, the instantaneous rotation center is located behind the intervertebral disc, the lower articular process on one side of the upper vertebral body of the injured segment passes through the upper articular process of the lower vertebral body to its anterior part, forming a unilateral articular process interlocking under the traction of the neck muscles and surrounding ligaments. Cervical intervertebral disc tear combined with vertebral body laterally shifted and unilateral facet joint locking is an unstable cervical spine injury. Most scholars believe that this situation must be ‘unlocked’ and reattachment first [5]. At present, the common methods of reattachment include manual traction reattachment, intraoperative detacher prying reattachment, TARP reset device assisted reattachment, combined with posterior surgical reattachment, and other schemes [1]. Direct manual traction reattachment is difficult due to muscle protective spasms of the posterior cervical muscle group. If combined with cervical fracture and dislocation or intervertebral disc compression, iatrogenic injury is likely to occur [6]. In the anterior cervical surgery, the periosteal stripper is used for vertebral body poking reduction. Because it depends entirely on the operator's hand feeling, the strength is not easy to grasp, which may cause spinal cord injury due to overstretch [7]. The use of a TARP reattachment device to assist reattachment requires temporary placement of fixed screws in the upper and lower vertebrae. When patients with vertebral fractures or osteoporosis are encountered, the screw-holding force will be greatly affected, and the reattachment effect is often poor. Although posterior surgery can directly deal with the reattachment of the articular process [8], however, it often means that the operation time is longer, the surgical wound is larger, and more the fixed segments. Some elderly patients may be difficult to tolerate surgery. Anterior surgery is still needed to deal with the anterior disc injury. Some Scholars compared the surgical efficacy and postoperative complications of anterior cervical surgery and posterior cervical surgery, and believed that the average amount of bleeding of the posterior cervical surgery was more than 15 ml the anterior cervical surgery, and the average reoperation rate of posterior cervical surgery was 2 % higher than the anterior cervical surgery [8]. Most scholars believe that skull traction can safely and effectively reattach the cervical facet joint locked, especially the unilateral ‘tip-to-tip’ facet joint locking is easier to reattach [7,9]. For this patient, we performed skull traction reattachment before surgery. The average traction weight was (6.0 ± 2.0) Kg, and the maximum weight of traction was 10 Kg. After continuous neutral traction, the facet joint was slightly flexed and then laterally flexed to the contralateral side to obtain satisfactory facet reattachment. Therefore, we believe that preoperative skull traction is a simple and effective way to reattach the method for unilateral ‘tip-to-tip’ facet joint locked, and this method has a high reattachment rate.

In addition, the injured intervertebral disc on preoperative MRI T2W1 showed a high signal, which was consistent with the changes of high signal in the disc and interspinous ligament injury signal on MRI in the cervical intervertebral disc injury considered by Kim et al. [10]. Following this, through the operation, we further clarified that the target intervertebral disc did have an injury. Davis et al. [11] followed up on the patients who underwent conservative treatment of intervertebral disc injury and found that there was still a high signal in the injured intervertebral disc 9 months after injury. Macnab [12] found that the intervertebral disc of the injured segment was easily peeled off from the bone endplate in patients who underwent surgery 2 years after the injury of the intervertebral disc, which means that the adhesion between the injured intervertebral disc and the endplate is poor, and the intervertebral disc has almost no repair ability. Other scholars [13,14] studies have shown that when the intervertebral disc cannot heal, it will accelerate the degeneration of the intervertebral disc. The degenerated will weaken the limitation of the range of motion of the adjacent vertebrae and lead to the relaxation of the surrounding ligaments, which will eventually aggravate the instability of the cervical spine. Biomechanical studies have shown that 80 % to 85 % of the axial load of the anterior column of the cervical spine [15], which means that when the intervertebral disc is damaged and torn, the stability of the cervical spine is destroyed, and the intervertebral disc cannot be repaired by itself, which makes anterior surgery necessary. Through the anterior cervical fusion surgery for patients with a cervical intervertebral disc injury, Ye Tianwen et al. [16] found that the fusion of the stable cervical spine can effectively avoid the secondary cervical spinal cord compression caused by cervical instability and intervertebral disc degeneration. After the reduction of the locked joint facet by skull traction, ACDF anterior surgery alone can obtain satisfactory mechanical support and stability reconstruction. Some scholars have compared simple anterior cervical surgery with combined anterior and posterior surgery for the treatment of cervical spine injury with facet joint locking and believe that the simple anterior approach also has a satisfactory therapeutic effect [17]. Not only because anterior surgery can directly relieve the compression of the cervical spinal cord caused by the tear of the nucleus pulposus and the intervertebral disc, but also through the preoperative skull traction, intraoperative distraction, and the placement of the fusion cage, the physiological curvature of the cervical spine and the height of the intervertebral space can be restored [18,19]. Neil Duggal [20] confirmed by biomechanical studies that the anterior approach titanium plate internal fixation and posterior approach internal fixation are equivalent in terms of stability, and can achieve better cervical spine stability reconstruction. Wang Weiming [21] treated more than 40 patients with lower cervical spine injuries by simple anterior approach and combined anterior and posterior approach respectively. It was found that both of them could better restore the Cobb angle of the cervical spine and the height of intervertebral space of injured vertebrae, and there was no significant difference in stability between the two groups.

Therefore, we believe that ACDF has the following advantages in the treatment of single-segment cervical disc tear without neurological symptoms combined with C2-C3 transverse dislocation and unilateral ‘tip-to-tip’ facet locked. (1) After skull traction reduction, the operation of anterior surgery alone can directly complete decompression, fusion, and internal fixation. (2) The operation of ACDF alone surgery is simple, and the operation has less trauma and fewer postoperative complications. (3) The operation of ACDF alone can directly deal with the torn intervertebral disc, avoiding cervical spinal cord injury caused by disc herniation. (5) There is no need to change surgical position during the operation, which reduces the possibility of secondary spinal cord injury. (5) The operation of anterior surgery alone has fewer fixed segments and can meet the requirements of cervical spine stability on reconstruction and biomechanics [24].

## 4 Conclusions

After successful reattachment by skull traction, elective operation of ACDF was treated on the patient with single-segment cervical disc tear combined with C2-C3 transverse dislocation and unilateral ‘tip-to-tip’ facet locked. We found that this scheme can not only directly deal with the torn intervertebral disc, but also can effectively complete the decompression of the spinal canal and stability reconstruction of the cervical spine. The patient had a small operative incision and fewer postoperative complications. The early clinical efficacy of the patient was satisfactory.

## Author contribution

Fan Yang(First Author): Conceptualization, Writing - original draft, Writing - review & editing.

Jiongbiao Zhong(Corresponding Author and Guarantor): Supervision.

Zheng Li: Writing - review & editing.

Peishan Wang: Writing - review & editing.

Jiarui Peng: Writing - review & editing.

Qing Quan: Writing - review & editing.

## Consent

Written informed consent was obtained from the patient for publication of this case report and accompanying images. A copy of the written consent is available for review by the Editor-in-Chief of this journal on request.

## Ethical approval

The study is exempt from ethical approval at the research institution.

## Guarantor

Jiongbiao Zhong(Corresponding Author and Guarantor)

## Research registration number

1.Name of the registry: Skull traction reattachment combined with ACDF in the treatment of C2-C3 transverse dislocation with unilateral cervical facet locked: A case report

2.Unique identifying number or registration ID: reviewregistry1944

3.Hyperlink to your specific registration (must be publicly accessible and will be

checked): https://researchregistry.knack.com/research-registry#user-systematicreviewmeta-analysesregistry/

## Sources of funding

Supported by Hunan Provincial Natural Science Foundation of China (2025JJ70250)

Supported by Hunan Provincial Natural Science Foundation of China (2020JJ9054).

Supported by the scientific research project of Hunan Provincial Health Commission (202104071123).

Supported by the Science and Technology Basic Research Guiding Project of Yueyang City (202012).

The study sponsor was involved in the collection, analysis and interpretation of part of the data, writing of the manuscript, and submission of the manuscript for publication.

## Declaration of competing interest

No conflicts of interest.

## References

[1] Li Y., Zhou P., Cui W. (2019). Immediate anterior open reduction and plate fixation in the management of lower cervical dislocation with facet locking. Sci. Rep..

[2] Brantigan J.W. (1993). Steffee a D.A carbon fiber implant to aid interbody lumbar fusion. Two-year clinical results in the first 26 patients. [J]. Spine.

[5] Nakashima H., Yukawa Y., Ito K. (2011). Posterior approach for cervical fracture-dislocations with traumatic disc herniation [J]. Eur. Spine J..

[6] Hoffmann RF, Weisskopf M, Stöckle U, Weiler A, Haas NP. Bisegmental rotational fracture dislocation of the pediatric cervical spine. A case report. Spine (Phila Pa 1976). 1999;24(9):904–907. doi:10.1097/00007632-199905010-00012. 10327513

[7] Rao Y., Li J., Liang S., Yang L., Han Z., Zhu B. (2021). Zhongguo Xiu Fu Chong Jian Wai Ke Za Zhi.

[8] Dominic T, Kleinhenz, Adam E M, Eltorai, Stephen, Huo et al. Traumatic cervical epidural hematoma due to fusion mass fracture following elective rod removal[J]. J. Orthop., 2017, 14: 0. 10.1016/j.jor.2017.07.014 PMC5574815 28878514

[9] Zhan P.L., Ye Z. (2009). Zhongguo Gu Shang.

[10] Tae Hun, Kim, Dae Hyun, Kim, Ki Hong, Kim et al. Can the zero-profile implant be used for anterior cervical discectomy and fusion in traumatic subaxial disc injury? A preliminary, retrospective study. [J] J Korean Neurosurg Soc, 2018, 61: 0. 10.3340/jkns.2018.0090 PMC6129748 30196654

[11] Davis S.J., Teresi L.M., Bradley W.G. (1991). Cervical spine hyperextension injuries: MR findings[J]. Radiology.

[12] Macnab L. (1964). Acceleration injuries of the cervical spine [J]. J Bone J Surg Am.

[13] Fakhoury J, Dowling TJ. Cervical degenerative disc disease. In: *StatPearls*. Treasure Island (FL): StatPearls Publishing; August 14, 2023. 32809607

[14] Barros E.M., Rodrigues C.J., Rodrigues N.R., Oliveira R.P., Barros T.E., Rodrigues A.J. (2002). Aging of the elastic and collagen fibers in the human cervical interspinous ligaments. Spine J..

[15] Zhou F., Zou J., Gan M., Zhu R., Yang H. (2010). Management of fracture-dislocation of the lower cervical spine with the cervical pedicle screw system. Ann. R. Coll. Surg. Engl..

[16] Ye T.W., Jia L.S., Chen X.S., Yuan W., Zhou X.H., Song D.W. (2006). Zhonghua Wai Ke Za Zhi.

[17] Zhu Y., Yue E., Kong Q. (2016). Zhongguo Xiu Fu Chong Jian Wai Ke Za Zhi.

[18] Lao L., Li Q., Zhong G. (2014 Nov 27). Biomechanical study of a novel self-locking plate system for anterior cervical fixation [J]. J. Orthop. Surg. Res..

[19] Lawrence B.D., Patel A.A., Guss A. (2014). Malaligned dynamic anterior cervical plate: a biomechanical analysis of effectiveness [J]. Spine.

[20] Duggal N., Chamberlain R.H., Park S.C., Sonntag V.K., Dickman C.A., Crawford N.R. (2005). Unilateral cervical facet dislocation: biomechanics of fixation. Spine (Phila Pa 1976).

[21] Chen J.M., Hu Y., Gu Y.J., Ma W.H., Xu R.M. (2010). Zhongguo Gu Shang.

